# Transcriptomic Characterization of the Human Habenula Highlights Drug Metabolism and the Neuroimmune System

**DOI:** 10.3389/fnins.2018.00742

**Published:** 2018-10-31

**Authors:** Bernard Le Foll, Leon French

**Affiliations:** ^1^Addictions Division, Centre for Addiction and Mental Health, Toronto, ON, Canada; ^2^Institute of Medical Science, University of Toronto, Toronto, ON, Canada; ^3^Department of Family & Community Medicine, University of Toronto, Toronto, ON, Canada; ^4^Department of Pharmacology & Toxicology, University of Toronto, Toronto, ON, Canada; ^5^Division of Brain and Therapeutics, Department of Psychiatry, University of Toronto, Toronto, ON, Canada; ^6^Campbell Family Mental Health Research Institute, Centre for Addiction and Mental Health, Toronto, ON, Canada; ^7^Krembil Centre for Neuroinformatics, Centre for Addiction and Mental Health, Toronto, ON, Canada

**Keywords:** transcriptomics, habenula, addiction, depression, neuroimmune, mast cell, drug metabolism, cannabis

## Abstract

Due to size and accessibility, most information about the habenula is derived from rodent studies. To better understand the molecular signature of the habenula we characterized the genes that have high expression in the habenula. We compared anatomical expression profiles of three normal adult human brains and four fetal brains. We used gene set enrichment analyses to determine if genes annotated to specific molecular functions, cellular components, and biological processes are enriched in the habenula. We also tested gene sets related to depression and addiction to determine if they uniquely involve the habenula. As expected, we observed high habenular expression of GPR151, nicotinic cholinergic receptors, and cilia-associated genes (medial division). Genes identified in genetic studies of smoking and associated with nicotine response were enriched in the habenula. Genes associated with major depressive disorder did not have enriched expression in the habenula but genes negatively correlated with hedonic well-being were, providing a link to anhedonia. We observed enrichment of genes associated with diseases that are comorbid with addictions (hematopoiesis, thrombosis, liver cirrhosis, pneumonia, and pulmonary fibrosis) and depression (rheumatoid arthritis, multiple sclerosis, and kidney disease). These inflammatory diseases mark a neuroimmune signature that is supported by genes associated with mast cells, acute inflammatory response, and leukocyte migration. We also found enrichment of cytochrome p450 genes suggesting the habenula is uniquely sensitive to endogenous and xenobiotic compounds. Our results suggest the habenula receives negative reward signals from immune and drug processing molecules. This is consistent with the habenular role in the “anti-reward” system and suggests it may be a key bridge between autoimmune disorders, drug use, and psychiatric diseases.

## Introduction

The habenula, located in the epithalamus, links midbrain and hindbrain regions to the forebrain. It’s considered to be at a crossroads, allowing it to act as a conductor of cognitive behaviors (Lecourtier and Kelly, 2007; Hikosaka et al., 2008). Landmark work in monkeys by Matsumoto and Hikosaka (2007) found that habenula neurons provide negative reward signals to midbrain dopamine neurons. Human functional neuroimaging results also find that the habenula encodes negative motivational value (Lawson et al., 2014). However, due to its small size and accessibility, most information about the habenula is derived from animal studies. Specifically, the habenular connections to the dopamine and serotonin systems; and its associations with motivation have spurred its study in animal models of depression and addiction. These studies have been reviewed in the context of psychiatric disease (Hikosaka, 2010; Proulx et al., 2014; Fakhoury, 2017), drug addiction (Lecca et al., 2014; Velasquez et al., 2014; McLaughlin et al., 2017) and withdrawal (Meye et al., 2017).

At the molecular level, gene expression images and profiles of the mouse and rat brain have been used to characterize the habenula (Wagner et al., 2016a,b). By determining which genes are specifically expressed in the habenula these studies have been able to define subregions and link molecular processes. In the human brain, transcriptomic profiling has been used to characterize genes differentially expressed in postmortem studies of depression, addiction, and other psychiatric diseases. However, these studies commonly focus on the frontal cortex or limbic regions (Mistry and Pavlidis, 2010; Colantuoni et al., 2011; Ding et al., 2015; Labonté et al., 2017). While the habenula has been linked to psychiatric disease, its transcriptional profile has not been analyzed in normal or diseased brains beyond a comparison of the 100 most specifically expressed genes (Boulos et al., 2017).

Large-scale efforts by the Allen Institute for Brain Science and their collaborators have led to the creation of genome-wide gene expression atlases of the human brain (Hawrylycz et al., 2012; Miller et al., 2014a). These atlases are anatomically comprehensive, providing transcriptomic profiles of over 200 regions that include the lateral and medial habenula. Unlike past studies of the human habenula which are limited by the resolution of MR imaging, this transcriptomic data enables analysis at the molecular scale.

In this present study, we used human transcriptomic data to determine which genes are specifically expressed in the lateral and medial habenula. We sought to learn if these genes were associated with addiction and depression relative to other brain regions. We also took a broader approach by assessing which cellular components, molecular processes, or functions and disease-associated genes were uniquely expressed in the habenula.

## Materials and Methods

### Adult Human Brain Gene Expression Data

Gene expression data that comprehensively covers the adult human brain was obtained from the Allen Human Brain Atlas (Hawrylycz et al., 2012). From the six brains assayed in the Atlas, we used only data from the three that had habenula samples (all male). Specifically, the lateral and medial habenula was acquired from two donor brains (ID:9861/H0351.2001, 24 years old; ID:12876/H0351.1009, 57 years old), and one with only a lateral sample (ID:15697/H0351.1016, 55 years old). Postmortem blood was tested for the presence of therapeutic drugs and drugs with abuse potential by the Allen Institute. Caffeine (all 3 donors), theobromine (2 donors), and atropine (1 donor) were detected in the donors at levels that are not considered toxicologically significant. The expression data were extensively annotated and normalized by the Allen Institute for Brain Science (adjusted for array-specific biases, batch, and dissection method). Data from the three donor brains contained 2,674 spatially resolved gene expression profiles, providing expression information for 217 unique named brain regions. Custom 64K Agilent microarrays were used to assay genome-wide expression. The 58,692 probes were filtered to 48,170 that mapped to a gene symbol in the Allen annotations. Details of the procedures used by the Allen Institute researchers are available in the Allen Human Brain Atlas technical white paper^1^.

### Prenatal Human Gene Expression Data

The BrainSpan consortium has created a similar transcriptomic atlas for the normal mid-gestational human brain (Miller et al., 2014a). The medial and lateral habenular nuclei were acquired in all four of the prenatal specimens used for this atlas (15–21 postconception weeks, 3 females and one male). These four specimens passed several exclusion criteria and no neuropathological defects were found by the consortium. Data from the four specimens contained 1,203 spatially resolved gene expression samples, providing expression information for 516 unique named brain regions. The same custom 64K Agilent microarrays that were used for the adult atlas were used to profile expression. Details of the procedures used by the Allen Institute researchers are available in the Brainspan Atlas of the Developing Human Brain technical white paper^2^.

### Region-Specific Expression Analysis

The limma software package was used to detect probes that are specifically expressed in the lateral and medial habenula (Ritchie et al., 2015). For each microarray probe, linear models were fit with coefficients for donor and region of interest for the lateral and medial habenula separately. In other words, expression of given probe across the complete set of expression measurements (adult: 2,674, fetal: 1,203) was predicted with a donor indicator variable and if the sampled region was the lateral and medial habenula (calculated separately). The empirical Bayes moderation method implemented in limma was used to calculate moderated t statistics and corresponding *p*-values (shrinks variances to a common value).

### Estimation of Cell-Type Proportion

The markerGeneProfile (MGP) R package was used to estimate cell type proportions from the expression profiles (Mancarci et al., 2017). This method uses the first principal component obtained from the expression of a set of cell-type specific markers to estimate the relative abundance of a cell type. For cell-type markers, we used genes from a study of healthy temporal cortex tissue that was obtained during resective surgery (Darmanis et al., 2015). This study provided the top 21 most enriched genes for astrocytes, neurons, oligodendrocytes, oligodendrocyte precursors, microglia and endothelial cell-types (via transcriptomic clustering, Supplementary Table S3; (Darmanis et al., 2015)). Default parameters for the mgpEstimate function were used with the geneTransform function set to the identity function. Proportions were estimated separately for each donor brain. Within a brain, proportions were mean averaged across multiple samples for each brain region. Regional proportions for each cell-type were then scaled and then mean averaged across the brains. Ranks were then computed across all regions for a specific cell-type to provide relative values of estimated proportions.

### Gene Set Enrichment Analysis

To summarize the probe level statistics we used the probe to gene mappings provided by the Allen Institute (Hawrylycz et al., 2012; Miller et al., 2014b). When many probes map to the same gene, we summarized the region-specific expression results by choosing the probe with the lowest *p*-value to represent the gene. For a given region of interest, *p*-values were combined with the direction of effect for the 20,779 genes (signed *p*-values). This provided a ranking from the gene with the most significant specific expression to the gene with the most significant depleted expression in the region. This genome-wide ranking allowed us to test if the genes that were specifically expressed in the habenula were enriched for a given gene set using the area under the receiver operator curve (AUROC) statistic. This non-parametric measure is frequently used for enrichment testing (Gillis et al., 2010). The AUROC for a set of genes is equivalent to the probability that a gene associated with that set will be found first in the genome-wide ranking compared to an unassociated gene. The ranking we used arranges genes from strongest specific to depleted expression. In this context, AUROC > 0.5 for a gene set means that these genes have specific expression in the habenula. For AUROC < 0.5 the reverse is true, with a bias toward depleted expression in the habenula. Given our focus on up-regulation, we only examined groups with AUROC > 0.5 (one-sided tests). AUROC values were generated using the tmod analysis package in R (Weiner and Domaszewska, 2016). The Mann–Whitney *U* test was used to determine statistical significance (one-sided). We used the Benjamini–Hochberg false discovery rate (FDR) procedure for multiple test correction (Benjamini and Hochberg, 1995) to adjust for the many tested gene sets. We performed no or limited multiple test correction for gene sets associated with depression and addiction. This *a priori* focus is marked by the inclusion of this article in the Frontiers Special Research Topic titled “Lateral Habenula: Role in Depression and Addiction”.

We also employ a specificity test to determine if the enriched gene sets are not representing broad differences that are significant across many other brain regions. For example, habenula specific gene sets may simply represent expression differences between the cortex and the rest of the brain. To assess specificity we ran the region-specific expression and gene set enrichment procedures for 516 fetal and 217 named brain regions in the expression datasets. For each gene set, we counted the number of regions with an AUROC value higher than the habenula result. Gene sets that have the highest specificity in the habenula subregions were considered to uniquely characterize the region.

### Gene Ontology Sets

The Gene Ontology (GO) classifies genes into sets associated with specific biological processes, molecular functions, and cellular locations to formally model biological systems (Ashburner et al., 2000). The GO database was accessed through the GO.db and org.Hs.eg.db packages in R (Carlson, 2017a,b). Annotations were dated March 29, 2017. We calculated the AUROC values for GO groups containing between 10 and 200 genes, after filtering for genes present in the Allen microarray data (6,623 GO groups annotating 14,530 unique genes).

### Disease-Associated Gene Sets

Disease-associated gene sets were obtained from the DisGeNET database which integrates several sources (Piñero et al., 2017). The curated gene-disease association file was downloaded in May 2018. Similar to the GO sets, we filtered for disease-associated gene sets with 10 to 200 genes after the intersection with our results (1,849 disease-associated gene sets covering 5,899 unique genes).

To complement the heterogeneous mix of sources in the DisGeNET we obtained genes from five large genetic studies focused on addiction or depression. For cannabis associated genes we used the seven genes harboring the genome-wide significant loci from the analysis of lifetime cannabis use [Table 1 in (Pasman et al., 2018)]. We additionally used the 35 genes that were significant in the gene-based analysis from Table 2 (genes found in the S-PrediXcan analysis only were excluded because they were linked to a specific tissue). The nine genes near the 14 genome-wide loci associated with alcohol consumption were obtained from Table 1 of Clarke et al. (2017). Genes associated with smoking were obtained from Minicã et al. (2017). Specifically, genes associated with ever smoking (3 genes), quantity smoked (14) and smoking cessation (2) were obtained from Table 1. Four genes linked to substance use disorder and five associated with opioid use disorder were obtained from Table 1 in a review of genetic studies by Jensen (2016). For depression, we used the 71 genes that harbor the 44 significant loci identified in the largest genetic study of major depression to date [Table 2 in (Wray et al., 2018)].

We additionally tested genes from transcriptomic studies of well-being and depression. These studies examined gene expression profiles of blood. The 27 genes from a study of recurrent major depression by Mostafavi et al. (2014) were split into up- and down- regulated lists (Table 2). For well-being, genes associated with hedonic well-being were obtained from Supplementary File D01 of Fredrickson et al. (2013).

### Availability

Scripts, supplementary tables, and data files for reproducing the analyses are available online at https://github.com/leonfrench/HumanHabenula and https://figshare.com/articles/Transcriptomic_characterization_of_the_human_habenula_highlights_drug_metabolism_and_the_neuroimmune_system/6756728.

## Results

### Lateral Habenula

**FIGURE 1:**
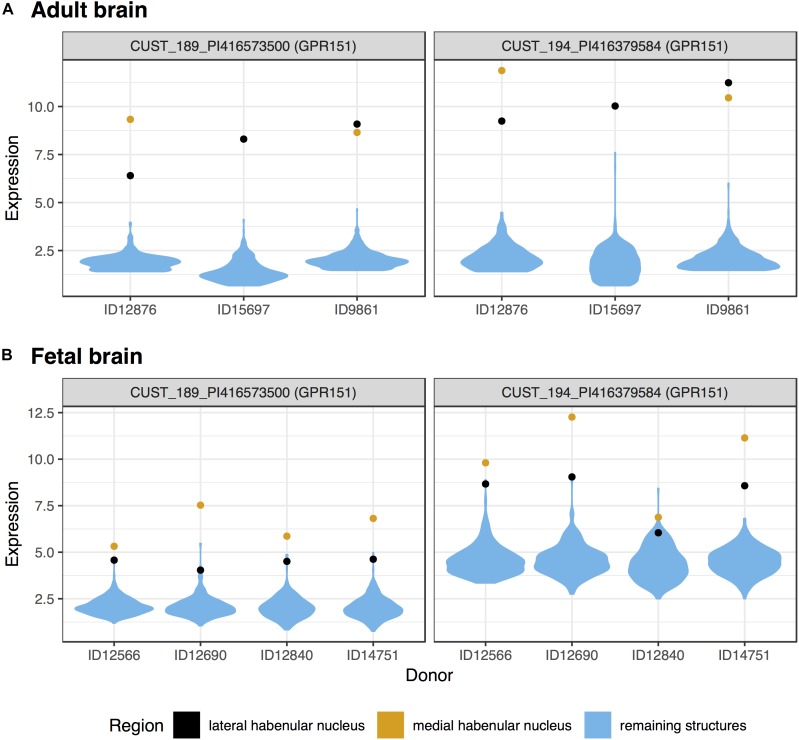
Violin plots of *GPR151* expression in the adult **(A)** and fetal **(B)** brains. Expression (log_2_ intensity) is plotted on the *y*-axis for each of the two probes for *GPR151*. Donor identification numbers are marked in the *x*-axis. Expression in the lateral habenula is marked in black with orange marking the medial division. The distribution of expression across the remaining sampled regions is shown in blue.

We first tested for specific expression in the lateral habenula in the adult brain data. Of the 48,170 tested microarray probes, 514 were significantly up-regulated in the lateral habenula (p_FDR_ < 0.05, Supplementary Table S1). These probes mapped to 405 genes, with the top 20 presented in Table 1. Both of the probes mapping to the most significant gene, GPR151, separate habenula from all other assayed structures (Figure 1A). In the fetal brain, 184 probes were significantly upregulated, representing 127 genes. Four genes were in both the fetal and adult top 20 most significantly up-regulated lists (*GPR151*, *POU4F1*, *ONECUT1*, and *CHRNB3*). Strong overlap was observed more broadly with 42 genes significantly up-regulated in both the fetal and adult expression profiles of the lateral habenula (hypergeometric test, *p* < 10^-39^).

**Table 1:** Top 20 genes with specific expression in the adult lateral habenula.

Gene symbol	Name	Significant probes	*p*-value
GPR151	G protein-coupled receptor 151	2	1.94E-85
MMRN1	Multimerin 1	2	1.61E-68
UGT2B15	UDP glucuronosyltransferase family 2 member B15	1	5.64E-36
WNT3A	Wnt family member 3A	1	3.87E-33
POU4F1	POU class 4 homeobox 1	2	6.61E-23
AQP2	Aquaporin 2	1	6.3E-22
PTCHD3	patched domain containing 3	1	1.21E-21
TRPM8	Transient receptor potential cation channel subfamily M member 8	2	2.45E-21
CYP3A5	Cytochrome P450 family 3 subfamily A member 5	1	4.55E-14
ONECUT1	One cut homeobox 1	2	4.62E-14
POU4F3	POU class 4 homeobox 3	1	4.08E-13
CPA1	Carboxypeptidase A1	1	1.63E-12
GNG8	G protein subunit gamma 8	2	1.61E-11
GALR1	Galanin receptor 1	2	2.16E-11
CHRNB3	Cholinergic receptor nicotinic beta 3 subunit	3	3.44E-11
ESM1	Endothelial cell specific molecule 1	2	4.32E-11
GPR15	G protein-coupled receptor 15	1	6.95E-11
ENO3	Enolase 3	1	3.07E-10
RCAN1	Regulator of calcineurin 1	2	1.06E-09
EGFR	Epidermal growth factor receptor	4	2.24E-09


### Medial Habenula

In the medial habenula, the 624 significant up-regulated probes mapped to 427 genes (p_FDR_ < 0.05, Supplementary Table S2). *GPR151* was the most specifically expressed gene in both the fetal and adult data for the medial division (Figure 1 and Table 2). In the fetal brain, 924 probes that map to 624 genes were upregulated in the medial habenula. In comparison to the lateral habenula, there was a stronger overlap between the adult and fetal results. Specifically, six genes overlapped within the top 20 lists (*GPR151*, *CYP3A4*, *CYP3A5*, *POU4F1*, *CHRNB3*, and *CAPN8*) and 160 were in both sets of significantly upregulated genes (hypergeometric test, *p* < 10^-132^).

**Table 2:** Top 20 genes with specific expression in the adult medial habenula.

Gene symbol	Name	Significant probes	*p*-value
GPR151	G protein-coupled receptor 151	2	1.74E-73
PDE11A	Phosphodiesterase 11A	5	1.62E-54
TRPM8	Transient receptor potential cation channel subfamily M member 8	2	3.09E-48
GNG8	G protein subunit gamma 8	2	1.1E-28
ESM1	Endothelial cell specific molecule 1	2	7.34E-26
CDC20B	Cell division cycle 20B	1	7.27E-23
CYP3A5	Cytochrome P450 family 3 subfamily A member 5	1	1.32E-21
CHRNB4	Cholinergic receptor nicotinic beta 4 subunit	2	2.85E-21
KLHDC4	Kelch domain containing 4	1	3.15E-21
WNT3A	Wnt family member 3A	1	3.25E-21
F13A1	Coagulation factor XIII A chain	2	2.8E-18
POU4F1	POU class 4 homeobox 1	2	4.48E-18
CYP3A4	Cytochrome P450 family 3 subfamily A member 4	1	8.49E-18
CYP3A7	Cytochrome P450 family 3 subfamily A member 7	2	3.51E-17
CHRNB3	Cholinergic receptor nicotinic beta 3 subunit	3	4.07E-17
POU4F3	POU class 4 homeobox 3	1	3.98E-16
SAA1	Serum amyloid A1	2	2.76E-15
CAPN8	Calpain 8	2	4.83E-15
ABCA13	ATP binding cassette subfamily A member 13	1	5.79E-14
ONECUT1	One cut homeobox 1	2	2.65E-12


### Cytochrome P450 Isoenzymes

**FIGURE 2:**
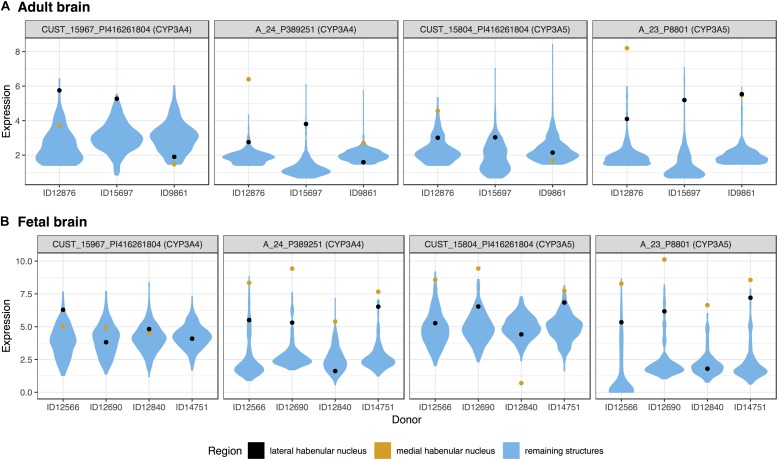
Violin plots of *CYP3A4* and *CYP3A5* expression in the adult **(A)** and fetal **(B)** brains. Expression (log_2_ intensity) is plotted individually for each probe on the *y*-axis (gene symbols in parenthesis). Donor identification numbers are marked in the *x*-axis. Expression in the lateral habenula is marked in black with orange marking the medial division. The distribution of expression across the remaining brain regions is shown in blue.

Surprisingly, three of the four members of the cytochrome P450 family 3 subfamily A, were in the top 20 most significantly up-regulated genes for the medial habenula (*CYP3A4*, *CYP3A5*, and *CYP3A7*; Table 2). *CYP3A4* and *CYP3A5* were also in the fetal top 20 (mapped probes are plotted in Figure 2). This finding is unique as none of the other 217 brain regions have 3 or more of the 61 cytochrome P450 genes in their top 20 lists (*CYP3A5* is in the lateral habenula top 20, adult data).

We extended this finding genome-wide by using the AUROC method to test the complete ranking of genes. Specifically, AUROC for the 61 cytochrome P450 genes was greater than 0.59 in the lateral and medial habenula (both *p* < 0.01). The four CYP3A subfamily genes had a stronger enrichment signal with AUROC above 0.85 in both divisions (*p* < 0.005). In the context of the other brain regions, only 5 other regions have higher AUROC values for the CYP3A genes. We named this count of brain-wide relative enrichment the “specificity rank” (lower is more specific). While the broad cytochrome P450 signal was not strong in the fetal data (both AUROC = 0.55, *p* > 0.08), the 3A subfamily genes were enriched in both divisions of the developing habenula (AUROC > 0.89, *p* < 0.005, specificity rank < 27). Across both divisions and datasets, *CYP3A4*, *CYP3A5*, and *CYP3A7* were consistently ranked within the top 227 most enriched genes while *CYP3A43* shows little enrichment (ranked within the top 6,444–11,744 genes). In summary, the CYP3A genes were specifically expressed in the fetal and adult habenula.

### Estimated Cell Type Proportions

We next used the markerGeneProfile tool to estimate cell-type proportions (Mancarci et al., 2017). These estimates were built from top marker genes obtained from a study of the adult human temporal cortex. Due to the difference in brain region, we only used the estimates to roughly characterize the cell-type composition of the habenula in the context of the other regions in the adult expression atlas. For both divisions, microglia had the highest estimated proportion with 15 and 18 regions having a higher estimate for the lateral and medial habenula, respectively. Astrocyte (specificity rank = 33) and endothelial cells (specificity rank = 35) also had above average estimated proportions the lateral habenula. In the medial habenula, endothelial cells were enriched (specificity rank = 58) with the remaining four cell types with above average estimated proportions (Supplementary Table S3).

### Addiction Focused Gene Set Enrichment Analyses

First, we tested GO terms related to drugs of abuse for enrichment in the adult habenula. Genes that were annotated to amphetamine, morphine, or cocaine response were not significantly enriched in the lateral or medial adult habenula (27–51 genes per group). The 47 genes annotated with the response to nicotine term were significantly enriched in the lateral (AUROC = 0.653, *p*_uncorrected_ < 0.00015) and medial habenula (AUROC = 0.614, *p*_uncorrected_ < 0.005). Brain-wide, this enrichment was specific with lateral habenula having the highest AUROC value of all 217 regions tested (specificity rank = 0). The medial finding was also relatively unique with a specificity rank of 10 (which includes the lateral habenula). Genes annotated to the response to alcohol term were significantly enriched in the medial (187 genes, AUROC = 0.54, *p*_uncorrected_ < 0.02, specificity rank = 30), but not the lateral habenula. In total, 12 GO terms have alcohol in their title. Of those terms, genes annotated to positive regulation of alcohol biosynthetic process were significantly enriched in both the medial and lateral habenula after correcting for the 12 tests (p_FDR_ < 0.005, AUROC > 0.67, specificity rank < 6). An additional two terms were enriched in the lateral but not medial habenula (‘regulation of alcohol biosynthetic process’ and ‘primary alcohol metabolic process’).

We next tested disease annotations related to addiction using the adult data. Of the eight gene sets with alcohol in their title, none were significantly enriched in the medial habenula and three had significant enrichment without correction for multiple testing in the lateral division (Non-alcoholic Fatty Liver Disease, Fetal Alcohol Spectrum Disorders and Fetal Alcohol Syndrome; *p*_uncorrected_ < 0.05 and p_FDR_ < 0.08). No significant enrichment was found for marijuana, cocaine or amphetamine abuse or dependence related gene sets.

To complement the disease associations from the DisGeNet database we tested genes from large genome-wide association studies of addiction. For addiction associated gene sets, none were significantly enriched after multiple test correction for the eight sets in the medial or lateral adult habenula data. For the lateral habenula, two genes associated with smoking cessation (*SLC25A21* [ranked 848], *SEMA6D* [ranked 1128], *p*_uncorrected_ < 0.02, AUROC = 0.95, specificity rank = 4), 14 associated with quantity of cigarettes smoked (*p*_uncorrected_ < 0.03, AUROC = 0.65, specificity rank = 13) and four genes associated with broad illicit drug use (*p*_uncorrected_ < 0.05, AUROC = 0.75, specificity rank = 3) were nominally enriched. The habenula specific genes associated with quantity smoked were cholinergic receptors (*CHRNA3* and *CHRNB4*) and major histocompatibility complex proteins (*HLA-DPA1* and *HLA-DPB1*). For the fetal lateral habenula, only the seven cannabis associated genes were enriched (*p*_uncorrected_ < 0.01, AUROC = 0.77, specificity rank = 12). For the medial division in the adult dataset, the four genes associated with broad illicit drug use were the most enriched and specific (*p*_uncorrected_ < 0.02, AUROC = 0.84, specificity rank = 0). The two smoking cessation associated genes, *SLC25A21* (ranked: 1021) and *SEMA6D* (ranked: 4285), were also enriched in the medial habenula data (*p*_uncorrected_ < 0.04, AUROC = 0.87, specificity rank = 20). These two sets were also enriched in the fetal data (*p*_uncorrected_ < 0.05 for both). The top-ranked set in the fetal medial data were the genes associated with quantity of cigarettes smoked (14 genes, *p*_uncorrected_ < 0.005, p_FDR_ < 0.04 [corrected for 8 sets], AUROC = 0.7, specificity rank = 6). Thus, some enrichment was observed for the genes harboring genetic variants associated with broad illicit drug use, cannabis, and smoking but not for alcohol consumption or opioid use.

### Depression Focused Gene Set Enrichment Analyses

**FIGURE 3:**
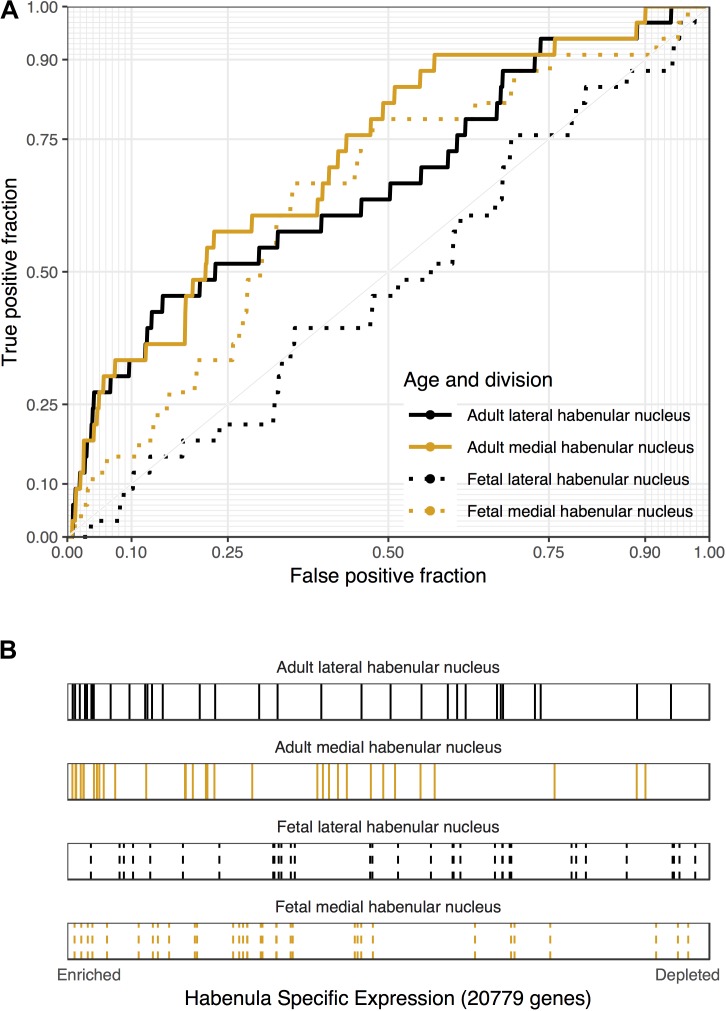
Associations between genes that are down-regulated in the blood of people with high levels of hedonic well-being and habenular specific expression. **(A)** ROC curves showing the proportion of hedonia down-regulated genes that overlap (*y*-axis, true positive fraction) in varying lengths of the habenula specific gene rankings (approximated by the *x*-axis, false positive fraction). **(B)** Distributions of the hedonia down-regulated genes across the habenular specific gene rankings with each gene representing a single colored line. Color marks the lateral (black) and medial (orange) habenula rankings. Dashed lines are used for the fetal datasets.

Of the four disease gene sets from DisGeNet that are related to depression, only “Depressive Symptoms” was significantly enriched for lateral habenula specific expression (29 genes, AUROC = 0.6, *p*_uncorrected_ < 0.05, specificity rank = 0). Genes harboring genetic loci that were associated with major depressive disorder in the largest genetic study to date were not enriched in the medial or lateral habenula (Wray et al., 2018). This enrichment for depressive symptoms and the central role of the habenula in the hedonic neurocircuit motivated an analysis of genes that were differentially expressed in a study of well-being (Graziane et al., 2018). Genes that were up-regulated in the blood of people with low levels of hedonic well-being were enriched in both divisions of the adult habenula (33 genes, *p* < 0.0005, AUROC > 0.67, specificity rank < 7, Figure 3). These genes that were negatively correlated with hedonic well-being were also enriched in the fetal medial habenula (*p* < 0.005, specificity rank = 1). In contrast, no significant enrichment was observed for genes up-regulated with hedonic well-being or genes differentially expressed in blood of individuals with recurrent major depression. Overall, we found enrichment for genes associated with depressive symptoms, including hedonic well-being but not genes linked to major depressive disorder.

### Gene Ontology Enrichment

We next used all the Gene Ontology annotations to obtain a broader characterization of the molecular functions, cellular components, and biological processes with high expression in the habenula. We applied the same procedures used for the above analyses with the addition of multiple test correction for the 6,623 GO groups used. For the lateral habenula, 981 groups were significant after correction and the top enriched GO groups cover a broad range of annotations (full list in Supplementary Table S4). The top result was the 196 genes annotated to the humoral immune response (p_FDR_ < 10^-11^, AUROC > 0.66, specificity rank = 3). Ten immune-related GO groups also appear within the top 15 with other groups representing peptidase regulation, glycosaminoglycan binding, odorant binding, and G-protein coupled peptide receptor activity (all p_FDR_ < 10^-7^, specificity rank < 13). For the medial habenula, 333 groups were significant after multiple test correction (full list in Supplementary Table S5). The most significant group was cilium movement (60 genes, p_FDR_ < 10^-10^, AUROC > 0.79, specificity rank = 4), which was joined by five other cilium or axoneme related gene sets in the top 15. This enrichment for cilia associated genes was expected because the medial habenula borders the 3rd ventricle. Within that top list, the remaining groups were immune (regulation of mononuclear cell proliferation and response to interferon-gamma) or nucleosome related (all p_FDR_ < 10^-5^, specificity rank < 37). Comparing the top 15 GO groups for the lateral and medial divisions revealed four shared immune-related groups: response to interferon-gamma, MHC protein complex, regulation of mononuclear cell proliferation and regulation of lymphocyte proliferation. Overall, stronger enrichment was found in the lateral habenula but this may be due to differing sample sizes (3 lateral versus 2 medial samples).

To filter the large number of significant GO groups, we selected groups that have a higher AUROC value than any other assayed region and were also enriched in the fetal samples (specificity rank = 0 and fetal *p*_uncorrected_ < 0.05). In the lateral habenula, applying the specificity leaves 114 of the 981 significant groups. As shown in Table 3, 38 groups were also enriched in the fetal brain samples (binomial test *p* < 10^-16^). While this list was primarily immune-related, acetylcholine binding and response to nicotine also appear. For the medial habenula, only four groups survive this filter (Table 4). To provide an extended list, we relaxed the specificity criteria to allow the gene group to have a higher AUROC value in one other brain region (specificity rank ≤ 1). Of the 31 groups with this specificity level, 22 were also significantly enriched in the fetal medial habenula at an *p*_uncorrected_ of 0.05 or lower (binomial test *p* < 10^-16^). These groups were primarily immune-related but also include two groups related to apoptosis via cysteine-type endopeptidase activity (Table 4).

**Table 3:** Gene Ontology (GO) groups enriched for uniquely specific expression in the adult lateral habenula which validate in the fetal brain.

Name	Genes	AUROC	Specificity rank	*p*-value_FDR_	Fetal AUROC	Fetal *p*-value
Glycosaminoglycan binding	200	0.643	0	2.21E-09	0.586	1.49E-05
Cytokine receptor activity	91	0.705	0	5.22E-09	0.598	0.000624
Leukocyte chemotaxis	188	0.641	0	7.17E-09	0.544	0.0195
Negative regulation of cell activation	162	0.643	0	7.24E-08	0.551	0.0126
Acute inflammatory response	129	0.658	0	9.44E-08	0.584	0.000486
Myeloid leukocyte migration	164	0.64	0	1.03E-07	0.539	0.0447
Positive regulation of epithelial cell proliferation	161	0.629	0	1.79E-06	0.544	0.0266
Positive regulation of chemotaxis	127	0.642	0	3.17E-06	0.566	0.00526
Negative regulation of cell-cell adhesion	137	0.633	0	6.69E-06	0.558	0.0101
Immunoglobulin binding	21	0.834	0	7.95E-06	0.671	0.00336
Mast cell activation	55	0.695	0	2.66E-05	0.571	0.034
Regulation of alpha-beta T cell activation	74	0.666	0	3.62E-05	0.581	0.00775
Regulation of acute inflammatory response	70	0.665	0	6.83E-05	0.591	0.00408
Alpha-beta T cell proliferation	27	0.764	0	7.46E-05	0.605	0.0297
Regulation of phosphatidylinositol 3-kinase signaling	145	0.61	0	0.000143	0.543	0.0363
Phosphatidylinositol 3-kinase signaling	163	0.602	0	0.000219	0.539	0.0414
Formation of primary germ layer	119	0.616	0	0.00032	0.556	0.0169
Positive regulation of lymphocyte differentiation	84	0.634	0	0.000483	0.576	0.0083
Regulation of humoral immune response	47	0.678	0	0.000549	0.575	0.0374
Negative regulation of leukocyte mediated immunity	44	0.681	0	0.000693	0.586	0.0245
Regulation of alpha-beta T cell proliferation	23	0.745	0	0.000896	0.614	0.0297
Acetylcholine binding	17	0.763	0	0.00225	0.69	0.00328
Icosanoid secretion	43	0.664	0	0.00243	0.592	0.0185
Collagen fibril organization	38	0.675	0	0.00243	0.592	0.0247
Response to nicotine	47	0.653	0	0.0033	0.571	0.0468
Anion:cation symporter activity	49	0.647	0	0.00393	0.596	0.00997
Positive regulation of mast cell activation	15	0.762	0	0.00429	0.637	0.0335
Peptide hormone processing	33	0.676	0	0.00458	0.617	0.0101
Acetylcholine-gated cation-selective channel activity	16	0.749	0	0.00515	0.625	0.0418
Branch elongation of an epithelium	20	0.721	0	0.00536	0.627	0.0242
Regulation of cell fate commitment	28	0.687	0	0.0054	0.614	0.0187
Acetylcholine receptor activity	22	0.711	0	0.00542	0.676	0.00215
Neurotransmitter transporter activity	25	0.696	0	0.00585	0.629	0.0126
Neurotransmitter:sodium symporter activity	19	0.72	0	0.00703	0.618	0.038
Regulation of T cell apoptotic process	32	0.666	0	0.00841	0.593	0.0348
Positive regulation of alpha-beta T cell differentiation	38	0.644	0	0.0128	0.589	0.0295
Neurotransmitter binding	22	0.673	0	0.0229	0.623	0.0229
Endodermal cell differentiation	45	0.615	0	0.0324	0.634	0.000942


**Table 4:** Gene Ontology groups enriched for uniquely specific expression in the adult medial habenula which validate in the fetal brain.

Name	Genes	AUROC	Specificity Rank	*p*-value_FDR_	Fetal AUROC	Fetal *p*-value
Positive regulation of leukocyte differentiation	132	0.639	1	6.56E-06	0.614	3.28E-06
Positive regulation of hemopoiesis	163	0.623	1	1.08E-05	0.588	5.15E-05
Regulation of B cell activation	108	0.625	0	0.000658	0.578	0.00268
Positive regulation of tumor necrosis factor superfamily cytokine production	59	0.666	1	0.000786	0.691	2.03E-07
Positive regulation of lymphocyte differentiation	84	0.634	1	0.00124	0.627	2.81E-05
Positive regulation of immune effector process	160	0.595	1	0.00167	0.592	2.86E-05
Cysteine-type endopeptidase activity involved in apoptotic process	15	0.787	1	0.00428	0.758	0.00027
Positive regulation of myeloid cell differentiation	80	0.622	1	0.00543	0.582	0.00574
B cell receptor signaling pathway	46	0.659	1	0.00603	0.623	0.00196
Microglial cell activation	18	0.753	1	0.00645	0.639	0.0204
Regulation of antigen processing and presentation	16	0.768	1	0.00645	0.706	0.00219
Positive regulation of myeloid leukocyte differentiation	50	0.65	1	0.0071	0.622	0.00142
Positive regulation of interleukin-6 production	66	0.626	1	0.0102	0.594	0.00422
Cysteine-type endopeptidase regulator activity involved in apoptotic process	43	0.654	1	0.0117	0.603	0.00972
Negative regulation of B cell activation	29	0.686	1	0.0125	0.609	0.0209
Positive regulation of CD4-positive, alpha-beta T cell activation	29	0.679	0	0.0166	0.661	0.00133
Positive regulation of cellular extravasation	13	0.76	0	0.0199	0.71	0.00433
Positive regulation of myotube differentiation	30	0.663	0	0.0288	0.595	0.0354
Granulocyte macrophage colony-stimulating factor production	15	0.727	1	0.0316	0.652	0.0206
Regulation of odontogenesis of dentin-containing tooth	13	0.742	1	0.0332	0.681	0.012
Positive regulation of CD4-positive, alpha-beta T cell differentiation	25	0.668	1	0.0406	0.665	0.00221
Positive regulation of adaptive immune response	80	0.591	1	0.0495	0.585	0.0044


### Disease-Associated Gene Enrichment

Beyond the addiction and depression sets of interest, several disease-associated gene sets ranked higher. Of the 1,849 tested sets, the top 10 most enriched in the lateral habenula are presented in Table 5 and include disorders that often result from addictions (Table 5, full list in Supplementary Table S6). In agreement with the gene ontology results, the top diseases for the medial habenula were primarily related to cilium dysfunction (Supplementary Table S7). Four diseases occurred in the top ten for both divisions (influenza, pneumonia, calcinosis and IgA glomerulonephritis).

**Table 5:** Top 10 disease-associated gene sets enriched for specific expression in the adult lateral habenula.

Name	Genes	AUROC	Specificity Rank	*p*-value_FDR_
Influenza	52	0.731	8	8.09E-06
Pneumonia	88	0.664	0	5.3E-05
Rheumatoid arthritis	172	0.616	0	6.21E-05
Calcinosis	45	0.716	0	0.000137
IgA glomerulonephritis	36	0.732	1	0.000285
Hypersensitivity	62	0.669	5	0.000661
Multiple sclerosis	38	0.698	2	0.00317
Uremia	17	0.789	0	0.00417
Pulmonary fibrosis	80	0.633	1	0.00417
Liver cirrhosis	130	0.604	2	0.00417


## Discussion

This study characterized the human lateral and medial habenula at the molecular scale. We tested for specific enrichment of genes associated with addiction and depression before performing a broader analysis. In agreement with a single-cell expression study of the larval and adult zebrafish habenula, our findings significantly overlapped between the adult and fetal results (Pandey et al., 2018). While focused on the adult habenula, we used the fetal data to validate key findings. Data from other brain regions was also used to determine specificity. In summary, we found the habenula specifically expressed genes associated with nicotine and smoking; cannabis; drug metabolism and clearance; acute immune response, mast cells, and inflammatory diseases; cilium; hedonic well-being; and hematopoiesis. We did not find strong or specific enrichment of genes associated with other drugs of abuse; learning, memory, and attention; nor major depressive disorder.

### Top-Ranked Genes

At the level of individual genes, we found the expected enrichment of cholinergic receptors with the nicotinic β3 and β4 subunits having the highest specificity in the lateral and medial division, respectively. Supporting translational potential, the β4 subunit was shown to limit nicotinic acetylcholine receptor activity and regulate nicotine aversion in studies of the mouse medial habenula (Frahm et al., 2011; Slimak et al., 2014). Combined, the 17 genes in the acetylcholine binding GO group that encode 14 nicotinic subunits, the muscarinic 3 receptor, acetylcholinesterase, and solute carrier family 18 member A3 (acetylcholine transporter) are specifically and uniquely enriched in the lateral habenula. However, the medial habenula had a higher number of significantly enriched cholinergic receptor genes after multiple test correction (nicotinic subunits α3, α4, α6, and β4). In the mouse medial habenula, receptors containing the α6 subunit were involved in nicotine withdrawal-induced anxiety (Pang et al., 2016). While habenular α5 subunit signaling has been shown to limit nicotine intake in rats, *CHRNA5* is not specifically expressed in the human habenula (Fowler et al., 2011). A recent mouse study suggests the α5 subunit may be important for nicotine preference and aversion in another region, the interpeduncular nucleus, which receives inputs from the medial habenula (Ables et al., 2017; Morton et al., 2018).

Confirming animal expression studies (Broms et al., 2015; Wagner et al., 2016a), the most specifically expressed gene in the habenula is a G protein-coupled receptor 151 (*GPR151*) which separated habenula expression from any other assayed region in the adult data. Similarly, specific expression of *GPR139* was observed, in agreement with rat and human expression patterns (Liu et al., 2015) (75th and 168th most specific gene the lateral and medial habenula, respectively). A recent study demonstrated that activation of GPR139 reduces alcohol intake in alcohol-dependent rats (Kononoff et al., 2018). Transient Receptor Potential Cation Channel Subfamily M Member 8 (*TRPM8*), was highly expressed in both the lateral and medial habenula (ranked 3rd and 8th in the medial and lateral divisions, respectively). This gene encodes a cation channel which is activated by menthol, providing a candidate molecule for studies examining the neurobiological effects of menthol on cigarette smokers (Gandhi et al., 2009; Brody et al., 2013; Henderson et al., 2016). Overall, the top-ranked genes confirm rodent studies and mark targets for future studies of addiction.

### Cytochrome P450 Enzymes and Drug Metabolism

Genes encoding cytochrome P450 enzymes were highly expressed in the medial habenula which has also been observed in rodent studies (Norris et al., 1996; Birnie et al., 2013). The metabolic functions of P450 enzymes are required to clear foreign and toxic compounds from the body. P450 substrates include therapeutic drugs, toxins, carcinogens, and some endogenous molecules that include testosterone and estrogen. For example, Cytochrome P450 Family 3 Subfamily A Member 4 (*CYP3A4*) is estimated to be involved in the metabolism of over half of all therapeutic drugs (Ioannides, 1996; Robertson et al., 2003). This is of note as *CYP3A4* was the 13th most specifically expressed gene for the adult medial habenula. For *CYP3A7*, we found more specific habenular expression in the adult when compared to the fetal data, which contrasts studies of the liver that found high fetal expression (Hakkola et al., 2001). In addition, UDP Glucuronosyltransferase Family 2 Member B15 (*UGT2B15*), the 3rd most specifically expressed gene in the lateral habenula, is also involved in the elimination of toxic compounds (xenobiotic and endogenous) (Stingl et al., 2014). Furthermore, *ADH7*, an alcohol dehydrogenase gene was enriched in the medial habenula (27th most specific gene). The combination of these enriched genes and the habenular border with the third ventricle suggest the habenula is uniquely sensitive to endogenous and xenobiotic compounds. This agrees with studies finding that the habenula is important for learning the adverse effects of drugs such as ethanol and nicotine (Fowler and Kenny, 2014; Haack et al., 2014). Beyond drugs of abuse, this association with drug metabolism may underlie links between therapeutic drugs and depression that are of increasing importance (Patten and Barbui, 2004; Qato et al., 2018).

### Mast Cells and Neuroimmune Associations

In the lateral habenula, genes annotated to mast cell activation were uniquely and specifically enriched as a set. Mast cells are first responders of the immune system, reside in the brain, and can cross the blood–brain barrier (Dong et al., 2014). Mast cells contain granules that can quickly release their stored contents in response to external stimuli. The contents of these granules are packed together with the help of glycosaminoglycans (Mulloy et al., 2017). Genes that bind glycosaminoglycan were the most significant and specifically enriched gene set in the lateral habenula. Mast cells respond to steroids and regulate the blood-brain barrier in the medial habenula of doves (Zhuang et al., 1996; Wilhelm, 2011). Mast cells react to xenobiotics, have been proposed as a paradigm for testing toxic effects of neurotropic drugs, and have been suggested to have detoxifying roles in the brain (Ibrahim, 1974; Purcell et al., 1996). More directly, habenular mast cells numbers are increased when rats are injected with cyclophosphamide, a toxic immunosuppressant and chemotherapeutic drug (Servière et al., 2003). This mast cell response was coupled with suppression of motor behavior. This key study connects our findings of enrichment of drug metabolism and mast cell associated genes with the habenula in an experimental setting.

Consistent with habenula being a source in negative reward signals, mast cells respond to aversive stimuli such as toxins, venoms, and physical stimuli (Galli et al., 2016; Kempuraj et al., 2017). Central nervous system mast cells are also associated with chronic subordination stress (Cirulli et al., 1998) and anxiety (Nautiyal et al., 2008). Patients with mastocytosis, a condition in which mast cells accumulate, are more likely to be depressed (Moura et al., 2011). A study of mastocytosis patients has also associated inflammatory markers with high depression scores (Georgin-Lavialle et al., 2016). In the context of addiction, naloxone-induced morphine withdrawal increases mast cell numbers in the thalamus (Taiwo et al., 2004) and intravenous heroin increases markers of mast cell degranulation (Rook et al., 2006). Overall, our findings suggest that the habenula is a key location for neuroimmune interactions (Forsythe and Bienenstock, 2012).

Beyond mast cells, we also observed enrichment of primarily immune-associated gene sets in Tables 3, 4. This can be partly explained by the relatively high estimated proportion of microglia cells. While 15 regions had higher estimated proportions of microglia these immune-associated gene sets were most strongly enriched in the habenula (specificity rank = 0), suggesting that these signals were not merely due to higher proportions of microglia. In agreement, a past study of the Interleukin 18 cytokine observed expression in medial habenula neurons (Sugama et al., 2002). One of most strongly enriched gene groups was ‘response to interferon-gamma,’ which is of interest given the recent associations between interferon-γ and social behavior (Filiano et al., 2016). Interferon-γ is also expressed in habenular neurons after influenza infection (Mori et al., 2001). Two other gene sets of note are ‘myeloid leukocyte migration’ and ‘positive regulation of cellular extravasation’ which supports findings of brain leukocyte infiltration from the cerebrospinal fluid into the habenula and other regions in rodents (Schmitt et al., 2012). More specific studies that have focused on pro-inflammatory molecules have associated the habenula with depressive symptoms and chronic restraint stress (Sugama et al., 2002; Zhao et al., 2018). Recent advances in single-cell sequencing may help determine which cell-types are contributing to this unique neuroimmune signature in the habenula.

### Depression

Genes associated with major depressive disorder that were identified in the largest genome-wide association study to date were not specifically expressed in the habenula (Wray et al., 2018). In addition, genes differentially expressed in the blood of individuals with recurrent major depression in comparison to controls (Mostafavi et al., 2014), were not specifically expressed in the habenula. Genes associated with depressive symptoms in the DisGeNet database were specifically and uniquely enriched in the lateral habenula. Also, genes negatively correlated with hedonic well-being were enriched in the medial and lateral habenula. This finding links the habenula to anhedonia, a key symptom of depression. Genes positively correlated with hedonic well-being were not enriched, suggesting the leukocyte source of these correlated genes was not driving the signal. In support, several studies have associated the habenula with hedonic pathways. In mice, the medial habenula was shown to regulate of hedonic state (Hsu et al., 2014). In patients with cancer-associated weight loss reduced functional connectivity was observed between the habenula and nucleus accumbens during a reward task (Maldonado et al., 2018). Differences in habenula volume has been associated with anhedonia symptoms in depressed and healthy individuals (Lawson et al., 2017). Habenula activation during a punishment task in unmedicated major depressive disorder patients was more strongly associated with anhedonia than overall depressive symptoms (Liu et al., 2017). Overall, we observed molecular associations with depressive symptoms and hedonic well-being but not genes directly linked to major depressive disorder.

Our broad analysis of disease-associated gene sets provided indirect links to depression. In the lateral habenula, we observed enrichment of several inflammatory diseases that align with the gene ontology analysis. In the context of depression, we note that that neuroinflammation has been associated with depression in mouse and human studies (Miller and Raison, 2016; Setiawan et al., 2018; Zhao et al., 2018). Genes associated with rheumatoid arthritis were the third most enriched set with no other region having a stronger association. A meta-analysis found that patients with rheumatoid arthritis have a higher prevalence of depression (Dickens et al., 2002). Similarly, genes associated with multiple sclerosis were enriched in the lateral habenula and depression is more common in multiple sclerosis patients (Patten et al., 2003). In addition, mast cells were found to be essential for disease progression in a mouse model of multiple sclerosis (Secor et al., 2000). IgA glomerulonephritis, an inflammatory kidney disease, was the 5th most enriched disease gene set. Genes associated with uremia were also specifically enriched (8th most enriched), which results from loss of kidney function. In patients with chronic renal disease, depression is the most common psychiatric condition (Fabrazzo and De Santo, 2006). Again, these results may be explained by a higher proportion of microglia in the habenula, but again we note that several other regions have a higher estimated number of microglia and these disease genes sets are very specific to the habenula. We also note that genes associated with influenza were top-ranked. Past studies have found that habenula neurons are selectively targeted by the influenza virus, resulting in behavioral changes after infection and clearance (Mori et al., 1999, 2001; Beraki et al., 2005). This suggests that neurons may underlie some of these inflammatory disease associations in the habenula. While these associations are interesting, more direct studies are needed to test the pathophysiological role of the habenula in these diseases.

### Addiction

Related to addiction and drugs of abuse we found limited specific expression in the habenula. We observed some enrichment of genes related to nicotine response and genetic studies of smoking behavior. Cannabis receptor 1 (*CNR1*) is significantly enriched in the adult lateral habenula and genes harboring genetic variants associated with cannabis use were enriched in the lateral division of the fetal brain but not the medial habenula in the adult or fetal data. In a rat model of nicotine addiction, habenular CB1 receptor activity in the habenula was linked to depressive-like behavior and recovery from nicotine dependence (Simonnet et al., 2017). In the context of nicotine, we note that the expression of molecules in regions that connect to the habenula play key roles. Specifically, rodent studies of glucagon-like peptide-1 in the nucleus tractus solitarius (Tuesta et al., 2017), dopamine D3 receptors in the basolateral amygdala (Khaled et al., 2014), and corticotropin releasing factor receptor-1 in the interpeduncular nucleus (Zhao-Shea et al., 2015) have linked nicotine intake, seeking, and withdrawal, respectively.

We found some habenular specific expression for genes involved in the regulation of alcohol biosynthesis but no enrichment of genes associated with alcohol consumption nor alcohol abuse. Also, we did not observe specific habenular expression of genes associated with amphetamine, morphine, or cocaine responses and related disorders. Past findings of increased habenula activity after administration of these drugs may be mediated by the cytochrome P450 enzymes. For example, the effect of cocaine on neurodevelopment was found to be mediated by *CYP3A5* (Lee et al., 2017). Also, the negative findings are possibly due to limited understanding of which molecules are involved in drug withdrawal and relapse, which the habenula is thought to play a key role (Velasquez et al., 2014; López et al., 2018).

In the context of addiction, we note that genes involved in the positive regulation of hemopoiesis are strongly enriched in the medial habenula. Hematopoiesis or hemopoiesis is the production of blood cells. A phenome-wide association study of clonal hematopoiesis has associated it with smoking, addiction and psychiatric disease (Zink et al., 2017). In addition, increases of several hematological factors which included cell type counts were observed in opium and heroin users (Haghpanah et al., 2010). A related result was enrichment of genes associated with thrombosis (12th most significantly enriched disease gene set in the lateral habenula). In the context of addiction, deep vein thrombosis is frequently caused by intravenous drug use (Cornford et al., 2011; Kwiatkowska et al., 2015). We also note that several of the lateral habenula disease-associated gene sets are associated with addictions: pneumonia [smoking and alcohol abuse (Grau et al., 2014)], liver cirrhosis (alcohol abuse), pulmonary fibrosis (smoking), arthritis (smoking), uremia and IgA glomerulonephritis [cocaine and alcohol abuse (Cecchin and De Marchi, 1996; Jaffe and Kimmel, 2006)]. Our analysis cannot determine the role, if any, played by the habenula or the enriched genes in the pathophysiology of these diseases or their associations with substance abuse. Nonetheless, these links between habenular gene expression, hemopoiesis, and the sequelae of addictions suggest complex relationships that warrant further investigation.

### Feeding and Energy Balance

An analysis of habenula specific expression in the rat brain highlighted genes associated with feeding and energy balance (Wagner et al., 2016a). Habenula activity has also been inversely associated with food hedonics in rodents (Kenny, 2011). In our gene set analysis, we did not observe enrichment of genes associated with eating behavior or energy regulation. However, we note that melanocortin 4 receptor (*MC4R*), was the 26th most significantly enriched gene in the lateral habenula. In addition, the five genes in the FTO obesity cluster were significantly enriched in the medial habenula which is primarily driven by the iroquois homeobox 5 and 6 genes (AUROC = 0.82, specificity rank = 14). Beyond *MC4R* and the FTO cluster, which were the two most significant genetic loci associated with body mass index, a larger set of genes harboring genetic variants associated with body mass index were not enriched in the habenula (Locke et al., 2015). Therefore, molecular studies of the habenula in the context of obesity should target the melanocortin system and iroquois homeobox genes.

## Conclusion

Numerous review articles have focused on the habenula but few mention the immune system or drug metabolism. Our results highlighted these underappreciated aspects of the region, suggesting the habenula is tuned to respond to negative signals from acute infection or xenobiotic compounds. This suggests that future studies of the human habenula should test effects of medication use. Our focused analysis highlighted genes associated with nicotine and cannabis but we did not observe associations with other specific drugs beyond a signal associated with broad substance abuse. It’s possible that habenular responses to these drugs may be mediated by the general drug metabolism genes that are enriched, or may be due to the limited understanding of the molecular mechanisms underlying withdrawal. Related to depression we observed an association with hedonic well-being but not broader depression. Across all diseases, we observed associations with inflammatory disorders which is mirrored by Gene Ontology analysis. While we estimate the habenula has an above average number of microglia, these specific neuroimmune associations are stronger and supported by animal studies. Overall, our results from the healthy brain suggest the habenula may be a key bridge to aid understanding of drug-induced depression and the role of inflammation in depression. More generally, our results suggest the habenula receives negative reward signals from immune and drug processing molecules. This is consistent with the habenular role in the “anti-reward” system.

## Author Contributions

BLF assisted in the interpretation of the data, manuscript writing, and editing. LF conceived the study, completed all analysis, interpreted the data, and drafted the manuscript.

## Conflict of Interest Statement

The authors declare that the research was conducted in the absence of any commercial or financial relationships that could be construed as a potential conflict of interest.

## Footnotes

1 http://help.brain-map.org/display/humanbrain/Documentation

2 http://help.brain-map.org/display/devhumanbrain/Documentation
